# *Strongyloides stercoralis* Dissemination and Hyperinfection Associated with Long-Term Steroid Treatment in a Neurosurgical Population

**DOI:** 10.1155/2023/4412935

**Published:** 2023-05-20

**Authors:** Mariana Constante, João Domingos, Francisco Neves Coelho, Teresa Baptista Fernandes, Teresa Baptista, Marta Maio Herculano

**Affiliations:** ^1^Egas Moniz Hospital, Ocidental Lisbon Hospital Center (Portugal), Department of Internal Medicine, Lisbon, Portugal; ^2^Egas Moniz Hospital, Ocidental Lisbon Hospital Center (Portugal), Department of Infectious Diseases, Lisbon, Portugal; ^3^Egas Moniz Hospital, Ocidental Lisbon Hospital Center (Portugal), Department of Intensive Care, Lisbon, Portugal; ^4^Egas Moniz Hospital, Ocidental Lisbon Hospital Center (Portugal), Department of Clinical Pathology and Microbiology, Lisbon, Portugal

## Abstract

Strongyloidiasis develops from the infection with *Strongyloides stercoralis* (Family: Strongylidae) and was recently considered a neglected tropical disease by the World Health Organization due to its global distribution and high burden of infection. Here, we present the cases of two patients under corticosteroid therapy after neurosurgical surgery who developed septic shock-like hyperinfection syndrome due to disseminated strongyloidiasis. The first case is a 77-year-old man from Cape Verde who was diagnosed with an extra-axial right parietal brain mass. He was given dexamethasone and was submitted to a biparietal craniotomy. His condition deteriorated and he was admitted to the intensive care unit (ICU), where he was diagnosed with disseminated strongyloidiasis with hyperinfection. Anthelmintic treatment and corticosteroid therapy were rapidly tapered and stopped. Neurological dysfunction persisted and the patient was transferred to the ward. The patient had died after complications of hospital-acquired pneumonia. The second case is a 47-year-old man from Guinea-Bissau who was diagnosed with a space-occupying lesion in the right temporal region and started treatment with dexamethasone. He underwent a craniectomy with partial excision of the lesion (high-grade glioma). Later his neurologic state worsened, and he was diagnosed with septic shock and hospital-acquired pneumonia. He was admitted to the ICU, the diagnosis of disseminated strongyloidiasis and hyperinfection syndrome was made and he initiated treatment with ivermectin and albendazole. Corticosteroid therapy was tapered. The patient's clinical status deteriorated, and multiple opportunistic infections were diagnosed during the ICU stay, which lead him to die. Clinicians should have a high index of suspicion when in the presence of corticosteroid-treated patients with sepsis. Preventive strategies and subsequent treatment should be considered in patients with a risk of acquisition or dissemination. Treating severe strongyloidiasis is still a clinical challenge and a delayed diagnosis can significantly worsen the outcomes of the patients affected, as seen in the presented cases.

## 1. Introduction

Strongyloidiasis develops from the infection with *Strongyloides stercoralis*, and to a lesser extent, the zoonotic *S. fulleborni*. *Strongyloides stercoralis* is a soil-transmitted intestinal helminth belonging to the family Strongylidae and was recently included in the World Health Organization's 2021–2030 road map for neglected tropical diseases [1] due to its global distribution and high burden of infection. Studies point to a prevalence as high as 370 million infected worldwide [2].

Tropical and subtropical areas such as Latin America, Southeast Asia, and sub-Saharan Africa seem to have a higher prevalence of infection, mainly due to soil contamination and warm and wet conditions facilitating human transmission, with some African countries reporting >90% global prevalence. Socioeconomic and cultural factors are determining the infection rate of a certain population and prevalence seems to be equivalent in males and females. There is, however, a lack of studies in many endemic countries (mainly in Africa) and high heterogeneity in the diagnostic methods used. In high-resource regions, such as Europe, most *S. stercoralis* infections are reported in rural areas, high-risk professions (farmers and miners), immigrants, refugees, and travellers to endemic regions [3].

*Strongyloides stercoralis* life cycle commonly begins with percutaneous infection by filariform larvae (considered as infective stage) present in contaminated soil, then migration to the lungs through blood circulation, ultimately reaching the gastrointestinal tract, where larvae molt twice and become adult female worms. Females live embedded in the submucosa of the small intestine and produce eggs via parthenogenesis (parasitic males do not exist), which yields rhabditiform larvae. Rhabditiform larvae can either be passed in the stool developing free-living cycles or become infective filariform larvae in the gut, which can penetrate either the intestinal mucosa or the skin of the perianal area, resulting in autoinfection. The parasite is therefore unique in its ability to maintain asymptomatic or oligo-symptomatic persistent infection in human hosts due to its auto-infective cycle [4]. Decade-long infections have been documented up to 75 years after exposure [5, 6].

Alcoholism, HIV and HTLV-1 infection, hematologic malignancies, post-transplant status, and other immunocompromising therapies are known risk factors for persistent *S. stercoralis* infection and also increase the risk of hyperinfection syndrome (HIS) and disseminated strongyloidiasis (DS) [3, 7, 8]. In HIS, host immunity dysfunction—particularly, impaired cell immunity—allows the uncontrolled proliferation of *S. stercoralis* and can facilitate the migration of larvae to organs outside the “classic” skin-lung-intestinal auto-infective cycle, a feature that is a characteristic of DS [4, 8]. Corticosteroid use is the most common factor associated with DS and HIS cases and both presentations have a high fatality rate, which can rise to 100% if no treatment is given [7].

Here, we present the cases of two patients under corticosteroid therapy after neurosurgical surgery who developed septic shock-like hyperinfection syndrome due to disseminated strongyloidiasis.

## 2. Case 1

A 77-year-old man with 4 months of progressive walking impairment went to a hospital emergency room at the end of September 2021. An extra-axial right parietal brain mass was diagnosed through a CT scan and the patient was admitted to the neurosurgery ward. The patient was born and lived in Cape Verde from 1944 to 1965. He moved afterward to urban areas in The Netherlands and then Portugal, where he worked for a merchant navy company, with occasional trips to the north, central, and occidental European coastal cities. He had no other known trips to tropical or subtropical countries. He was previously diagnosed with hypertension and dyslipidemia and was otherwise healthy.

Due to the presence of vasogenic edema, the patient was given therapy with 12 mg of dexamethasone daily since the first day of admission. The patient was submitted to a biparietal craniotomy in mid-October and an excision of the brain mass. He was clinically improving until about 10 days later when a Gram-negative microorganism (*Klebsiella pneumoniae*) was detected in multiple blood cultures collected on various days and targeted antibiotic therapy was initiated.

His condition deteriorated with fever, lethargy, severe hypoxia, and septic shock, and he was admitted to the intensive care unit (ICU) at the beginning of November. At admission to the ICU, he presented with a chest radiography compatible with bilateral pneumonia. Orotracheal invasive mechanical ventilation and vasopressor therapy were initiated.

**Figure 1:**
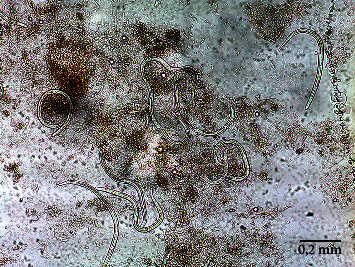
*Strongyloides stercoralis* larvae in an unstained wet mount of stool. 100x magnification (field of vision 2 mm).

The sputum and stool microscopic direct examination identified a high number of *Strongyloides stercoralis* filariform and rhabditiform larvae (Figure 1) and a parasitological urine examination was also positive for *S. stercoralis* larvae. A lumbar puncture was performed with normal cerebrospinal fluid (CSF) analysis (two cells/*μ*L, protein 44 mg/dL, glucose CSF/serum ratio >50%). No microorganisms were identified in direct visualization (including *S. stercoralis* larvae); however, the polymerase chain reaction (PCR) was positive for *S. stercoralis* nucleic acids. No cutaneous lesions were observed, and the patient had no eosinophilia. HTLV-1/2 and HIV-1/2 serologies were negative. The diagnosis of disseminated strongyloidiasis with hyperinfection was made. Treatment with ivermectin (200 *μ*g/kg/day) via nasogastric tube was initiated and albendazole (400 mg bid) was added two days later. Corticosteroid therapy was rapidly tapered and stopped after seven days.

During the ICU stay, he was later diagnosed with ventilator-associated pneumonia due to *Klebsiella pneumoniae* carbapenemase (KPC)-producing and meningitis due to *Enterococcus faecium,* which were treated accordingly. A cerebral mass biopsy revealed a World Health Organization grade II meningioma with bone and dura mater invasion.

There was a significant hemodynamic and respiratory improvement with antibiotics and antiparasitic. Neurological dysfunction, however, persisted after suspension of all sedative-analgesic medication and the patient remained comatose. Brain CT scan and MRI revealed only findings consistent with the previous surgical procedure and no cerebral parenchyma changes. Electroencephalography showed a persistent burst suppression pattern with no paroxysmal activity, suggestive of severe encephalopathy. There was no neurological improvement during the ICU stay and a tracheostomy was performed.

He was transferred to the ward after a total of 30 days in the ICU. The patient's stool and sputum samples became both negative for *S. stercoralis* larvae after 23 days of therapy and antiparasitic were maintained for an additional 20 days. The patient had died after complications of hospital-acquired severe acute respiratory syndrome coronavirus 2 (SARS-CoV-2) pneumonia. No autopsy was performed.

## 3. Case 2

A 47-year-old man went to the emergency room at the end of September with a severe headache. A space-occupying lesion in the right temporal region with severe vasogenic edema was diagnosed through a CT scan. He was admitted to the neurosurgery ward, where he initiated therapy with 12 mg of dexamethasone daily.

He was born in Guinea-Bissau in 1974 having lived for variable periods in both that country and in Portugal. He worked in private security and had no previous occupation that involved contact with soil. He had no other known trips to tropical or subtropical countries. He was in remission of colon adenocarcinoma, and previously submitted to a hemicolectomy in 2014 and adjuvant chemotherapy. He also had the diagnosis of chronic kidney disease after traumatic nephrectomy and serologic markers of previous hepatitis B infection (negative AgHBs, AgHBe, AcHBs, HBV DNA viral load <10 UI/mL, and positive AcHBe and AcHBc).

In mid-November, the patient underwent a craniectomy with partial excision of the lesion and the extemporaneous histologic examination revealed a high-grade glioma. He was globally improving and walking autonomously in the ward until the fourth day after surgery when his neurologic state worsened suddenly, and tonic-clonic seizures were observed. Brain CT scan revealed no changes and seizures subsided after increasing antiepileptic drug doses. Six days later, he developed fever, hypotension, and dyspnea. Chest X-rays revealed bilateral diffuse infiltrates. He was diagnosed with septic shock and hospital-acquired pneumonia with worsening respiratory failure. He was admitted to the ICU 10 days after the surgery invasive mechanical ventilation, vasopressor therapy, and broad-spectrum antibiotics were initiated. Kidney function deteriorated, and the patient commenced continuous hemodiafiltration.

**Figure 2:**
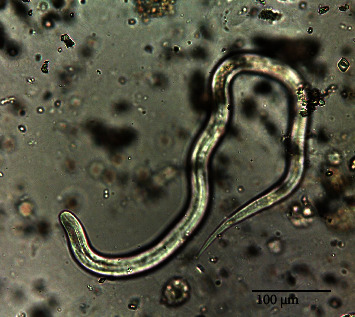
*Strongyloides stercoralis* filariform larvae in an unstained urine sample. 400x magnification (field of vision 450 *μ*m).

The urine pneumococcal antigen test was positive and *Klebsiella pneumoniae* was isolated in blood cultures. A chest CT scan showed bilateral pneumonia and findings compatible with acute respiratory distress syndrome. The microscopic direct examination of the sputum (and latter urine) revealed *S. stercoralis* larvae (Figure 2). Lumbar puncture was performed and revealed a normal CSF analysis (<1 cells/*μ*L, protein 36 mg/dL, glucose CSF/serum ratio >50%), and no microorganisms were identified using indirect visualization and culture. CSF PCR was negative for *S. stercoralis* nucleic acids. No cutaneous lesions or eosinophilia were present. The diagnosis of disseminated strongyloidiasis and hyperinfection syndrome was made and treatment with ivermectin (200 *μ*g/kg/day) was initiated via nasogastric tube and albendazole (400 mg bid) (D41). HTLV-1/2 and HIV-1/2 serologies were negative. Corticosteroid therapy was tapered and eventually stopped.

The patient was under vasopressor therapy for several days due to severe septic shock and experienced prolonged gastrointestinal stasis even under prokinetic agents. Three days after the diagnosis of disseminated strongyloidiasis and hyperinfection syndrome, a formal request was made to the Portuguese National Authority of Medicines and Health Products (INFARMED) for the off-label administration of the subcutaneous ivermectin (veterinary formulation). This subcutaneous form of ivermectin (1%) was made available 10 days after the diagnosis and administration was started on the same day in the dose of 200 *μ*g/kg/day divided by two subcutaneous injections given in different areas.

Meanwhile, the patient's clinical status worsened considerably. Multiple opportunistic infections were diagnosed during the ICU stay, namely, hepatitis B virus reactivation (AgHBs seroconversion and positive HBV DNA viral load 29 UI/mL), cytomegalovirus reactivation (CMV viral load 3934 IU/mL) with possible colitis, *Aspergillus fumigatus* spp. complex pulmonary aspergillosis, invasive candidiasis due to *Candida albicans* (bloodstream infection) and severe herpes simplex virus type 1 (HSV-1), and varicella-zoster virus (VZV) oral mucositis. Targeted antibiotic, antiviral, and antifungal therapies were initiated with little clinical response. The patient died after 17 days in the ICU. No autopsy was performed.

## 4. Discussion

Both cases described here are examples of *S. stercoralis* disseminated infection and hyperinfection syndrome, both rare conditions that occur more commonly in patients with immunocompromising conditions or undergoing immunocompromising therapy. Both patients had a recent diagnosis of a brain mass, and both had been taking a high dose of dexamethasone for several weeks when the diagnosis of *S. stercoralis* infection was made. Corticosteroid therapy appears to have been the triggering event for dissemination for both cases and neither patient had previous screening or preemptive treatment for strongyloidiasis before the start of immunosuppression. A summary of both cases is presented in Table 1.

When considering the place of exposure to the parasite, both patients were born in countries (Cape Verde and Guinea-Bissau) where strongyloidiasis is endemic [3, 9, 10] and probably acquired the infection while living in their respective home countries, maintaining a carrier state through low levels of autoinfection.

Portugal is not usually considered a highly endemic country although some cases of autochthonous infection were described during and until the end of the 20^th^ century when it is presumed that poorer sanitary conditions maintained some level of endemic transmission. Most cases were reported in the central west region of continental Portugal and in the archipelago of Madeira, where particular climatic and soil conditions contribute to the survival chance of parasitic larvae in soil [11]. Likewise, the estimated prevalence of strongyloidiasis in The Netherlands is reported to be very low and affects only travellers or overseas military personnel [10, 12].

In the first case described, the patient was last in Cape Verde 56 years before the diagnosis, and it can be assumed that he was infected for several decades before manifesting as DS and HIS. This finding is consistent with other cases described in the literature in which the onset of symptomatic strongyloidiasis occurred decades after initial exposure [5, 6]. In the second case, the patient regularly travelled to Guinea-Bissau, so recent and recurrent exposures cannot be excluded.

Both cases presented features of septic shock with bloodstream infection caused by Gram-negative bacteria. The mechanism of sepsis in patients with *Strongyloides* infection is thought to be through the translocation of enteric bacteria by filariform larvae when moving through the bowel wall [4]. Sepsis due to Gram-negative bacilli with bacteraemia and other organ involvement (mainly pneumonia and meningitis) is a frequent presentation described in DS/HIS [8], including corticosteroid-treated patients [13], and also seen in both of our cases.

Disseminated strongyloidiasis affecting the brain and meninges is rare, but some cases have been described in the literature sometimes only with postmortem diagnosis; in some cases, central nervous system (CNS) invasion is documented antemortem by isolation of *S. stercoralis* larvae in CSF or brain [14–19]. In our first case, even though no distinct larval forms were observed in CSF samples, CNS involvement was presumed due to PCR detection of *S. stercoralis* nucleic acids in the patient's CSF and unexplained prolonged encephalopathy.

Chronic nonsevere strongyloidiasis treatment is mostly based on the use of oral ivermectin or benzimidazoles (albendazole and thiabendazole), with ivermectin showing higher or comparable rates of cure and better adverse effects profile [20]. A new drug, moxidectin, is being evaluated as a promising alternative to the treatment of helminth infections, including strongyloidiasis [21]. In patients with severe disease, oral absorption may be impaired due to obstructive/paralytic ileus or changes in pharmacokinetic conditions. Rectal enemas or subcutaneous administration of veterinary formulations of ivermectin have been described as a possible alternative to oral formulations, but the doses used are variable [22]. In the second case reported here, a special authorization was submitted to Portugal's national drug regulatory agency to acquire and use a parenteral ivermectin veterinary formulation to bypass perceived oral absorption failure due to gastrointestinal ileus. Unfortunately, the subcutaneous formulation of ivermectin was only made available to the clinical team 10 days after the diagnosis and 5 days before the death of the patient.

Fatality rates in severe cases (DS and/or HIS) were very high (63%), even when adequate treatment was given. In some case reports, combination therapy was used, but an optimal treatment regimen is yet to be established [7]. In the first case presented the response to treatment and the cure of *S. stercoralis* HIS was assumed after the absence of larvae in biologic samples for two weeks (time required for a full cycle of *S. stercoralis* autoinfection). Both of our patients had several concurrent complications during their ICU stay and both had unfavourable outcomes; the first patient stayed in a coma and died before hospital discharge and the second had several opportunist infections which lead to death before the *S. stercoralis* infection was deemed cured.

Clinicians should have a high index of suspicion when in the presence of corticosteroid-treated patients with sepsis, with or without gastrointestinal symptoms. Preventive strategies, such as screening for chronic infection (by repeated stool examination) or exposure (serology), and subsequent treatment should be considered in patients with a risk of acquisition or dissemination. Presumptive treatment is possibly a life-saving alternative strategy for patients undergoing immunosuppression with a high probability of exposure, namely, those who travel or lived in high-endemic regions [23].

Treating severe strongyloidiasis is still a clinical challenge and a delayed diagnosis can significantly worsen the outcomes of the patients affected, as seen in the presented cases.

## Figures and Tables

**Table 1:** A summary and comparison between the two clinical cases.

	Case 1	Case 2
Age (years)/gender	77/male	47/male

Possible/probable exposure Date/timing of exposure	Cape Verde From 1944 to 1965	Guinea-Bissau From several years to months before

Risk factors for infection and HIS/DS (3, 7, 8)		
Alcoholism	No	No
HIV-infection	No	No
HTLV-1 infection	No	No
Hematologic malignancy	No	No
Solid organ transplant	No	No
Immunosuppressive therapy	Yes	Yes
Type	Corticosteroid (dexamethasone)	Corticosteroid (dexamethasone)
Cumulative dose equivalence (prednisone) until HIS/DS	1426 mg	3067 mg
Time from beginning immunosuppression to HIS/DS	32 days	37 days

Other comorbidities	Hypertension Dyslipidaemia	Colon adenocarcinoma in remission Chronic kidney disease Previous hepatitis B

Confirmed organ/system involvement during DS	Gastrointestinal Pulmonary Urinary Central nervous system	Gastrointestinal Pulmonary Urinary

Strongyloidiasis therapy given	Ivermectin 200 *μ*g/kg id (oral, NGT) Albendazole 400 mg bid (oral, NGT)	Ivermectin 200 *μ*g/kg id (oral, NGT) 10 days Ivermectin 200 *μ*g/kg id (subcutaneous) 5 days Albendazole 400 mg bid (oral, NGT) 15 days

Complications	(i) Bacteraemia due to *K. pneumoniae* (ii) Ventilator-associated pneumonia due to KPC-producing *K. pneumoniae* (iii) Meningitis to *E. faecium*	(i) CMV reactivation with possible colitis (ii) Pulmonary aspergillosis (iii) Bacteraemia due to *K. pneumoniae* (iv) Bloodstream infection due to *C. albicans* (v) HSV-1/VZV oral mucositis (vi) HBV reactivation

Outcome and time to outcome (after diagnosis)	Cure of strongyloidiasis in 4 weeks Death after 61 days (due to hospital-acquired SARS-CoV-2 pneumonia)	No cure for strongyloidiasis Death after 15 days

Note: CMV: cytomegalovirus; DS: disseminated strongyloidiasis; HBV: hepatitis B virus; HIS: hyperinfection syndrome; HIV: human immunodeficiency virus; HSV-1: herpes virus simplex 1; HTLV-1: human T-lymphotropic virus 1; KPC: *Klebsiella pneumoniae* carbapenemase; NGT: nasogastric tube; SARS-CoV-2: severe acute respiratory syndrome coronavirus 2; VZV: varicella zoster virus.

## Data Availability

The data supporting this case report are from previously reported cases, studies, and datasets. The processed data are available from the corresponding author upon request. These prior studies (and datasets) are cited at relevant places within the text as references [1–23].
